# 3D Interfacial and Spatiotemporal Regulation of Human Neuroepithelial Organoids

**DOI:** 10.1002/advs.202201106

**Published:** 2022-06-06

**Authors:** Chunling Tang, Xinhui Wang, Mirko D'Urso, Cas van der Putten, Nicholas A. Kurniawan

**Affiliations:** ^1^ Department of Biomedical Engineering Eindhoven University of Technology PO Box 513 Eindhoven 5600 MB The Netherlands; ^2^ Institute for Complex Molecular Systems PO Box 513 Eindhoven 5600 MB The Netherlands

**Keywords:** cellular self‐organization, dorsal–ventral patterning, interfacial cues, neural tube, neuroepithelial organoid

## Abstract

Neuroepithelial (NE) organoids with dorsal–ventral patterning provide a useful three‐dimensional (3D) in vitro model to interrogate neural tube formation during early development of the central nervous system. Understanding the fundamental processes behind the cellular self‐organization in NE organoids holds the key to the engineering of organoids with higher, more in vivo‐like complexity. However, little is known about the cellular regulation driving the NE development, especially in the presence of interfacial cues from the microenvironment. Here a simple 3D culture system that allows generation and manipulation of NE organoids from human‐induced pluripotent stem cells (hiPSCs), displaying developmental phases of hiPSC differentiation and self‐aggregation, first into NE cysts with lumen structure and then toward NE organoids with floor‐plate patterning, is established. Longitudinal inhibition reveals distinct and dynamic roles of actomyosin contractility and yes‐associated protein (YAP) signaling in governing these phases. By growing NE organoids on culture chips containing anisotropic surfaces or confining microniches, it is further demonstrated that interfacial cues can sensitively exert dimension‐dependent influence on luminal cyst and organoid morphology, successful floor‐plate patterning, as well as cytoskeletal regulation and YAP activity. This study therefore sheds new light on how organoid and tissue architecture can be steered through intracellular and extracellular means.

## Graphical abstract

The formation of human cystic neuroepithelial (NE) organoids can be induced in a simple yet manipulatable 3D culture system. Single pluripotent stem cells undergo self‐aggregation and differentiation, lumen formation and polarization, and finally form floor‐plate patterned NE organoids. These developmental phases are shown to be sensitive to interfacial cues, providing a new avenue to modulate organoid formation and maturation.

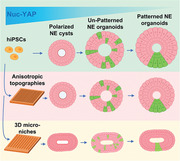

## 1 Introduction

Cellular patterning of the neural tube (NT) along the dorsal–ventral (DV) axis is a critical yet complex step during the neurulation stage of early embryogenesis.^[^ ^1^, ^2^, ^3^ ^]^ This patterning leads to the generation of different neuronal progenitor cells and the development of the central nervous system. Three‐dimensional (3D) neural epithelial (NE) organoids derived from pluripotent stem cells or embryonic stem cells are ideal model system to study the emergence of multicellular tissue complexity, as they have been shown to recapitulate several key features of in vivo development during neurulation stage.^[^ ^4^, ^5^, ^6^ ^]^ In fact, NT‐like patterning with roof plate and floor plate along the DV axis can be reassembled in NE organoids. NE organoids are commonly generated through self‐aggregation of stem cells to form organized 3D architecture and functions in reconstituted extracellular matrices.^[^ ^4^, ^5^, ^6^, ^7^ ^]^ While the role of inductive and morphogenetic factors as well as the biochemical signaling networks that underlie the cell differentiation in this process have been extensively studied,^[^ ^6^, ^7^ ^]^ how the self‐organization and cellular patterning in NE organoids take place in the presence of extracellular interfacial cues is still largely unknown. This has hampered the rational design of next‐generation NE organoid models with more complex cellular organizations that can better mimic the complete NT developmental process.

Exciting works in the last few years have started to explore bioengineering approaches to present specific physical extracellular cues for guiding organoid self‐organization and cell‐fate determination.^[^ ^7^, ^8^, ^9^, ^10^ ^]^ In particular, substrate geometries were used as artificial physical boundaries during cellular and tissue organization, enabling shape‐guided organoid morphogenesis.^[^ ^8^, ^9^, ^10^ ^]^ For instance, 3D intestinal organoids grown in tubular microniches organize into crypt‐like structures, following the predefined shape.^[^ ^8^, ^10^ ^]^ Previous studies of NE organoids using a poly(ethylene glycol) hydrogel‐supported 3D culture system reported that both the formation and the DV patterning of NE organoids could be modulated by extracellular matrix rigidity.^[^ ^6^, ^7^ ^]^ These studies not only point to the potential of using extracellular cues to modulate the formation of NE organoids, but also suggest the involvement of cell's intracellular organization and sensing of the physical environment. Indeed, significant actin cytoskeletal rearrangement that stabilized into a central actin ring has been detected during NE cyst formation.^[^ ^6^ ^]^ Actin and myosin assembled into contractile actomyosin bundle or stress fibers in nonmuscle cells, whose dynamics are generally known to be important for a variety of cell behaviors, including migration, aggregation, and construction of multicellular structures.^[^ ^11^, ^12^, ^13^ ^]^ Moreover, cytoskeletal organization and the Hippo signaling pathway, which is important in determining cell fate, are highly mechanosensitive and dependent on extracellular cues.^[^ ^14^, ^15^, ^16^, ^17^ ^]^ Both cytoskeletal arrangement and activity of yes‐associated protein (YAP) respond to extracellular geometrical cues, including surface topographies and confinement.^[^ ^14^, ^15^, ^16^, ^17^ ^]^ In the context of NT development and NE organoid formation, the roles of these intracellular regulation and extracellular cues remain poorly understood.

In this study, we present a simple yet manipulatable 3D culture system to produce cystic NE organoids. With this approach, human‐induced pluripotent stem cells (hiPSCs) undergo self‐aggregation and differentiation, lumen formation, and polarization to form NE cyst, which were then patterned with roof plate and floor plate along the DV axis to form NE organoids. We further show that the organization of the actin cytoskeleton and the activation of YAP are required and play differential roles in modulating different phases of NE organoid self‐organization. Moreover, extracellular interfacial cues in the form of soluble ligands, anisotropic topographies, and geometrical confinement in microfabricated substrates and 3D microniches were found to sensitively control the morphogenetic process and cellular patterning in the NE organoids. Together, our study reveals how interfacial cues spatiotemporally regulate and can be used to modulate 3D cellular self‐organization in human NT development.

## 2 Results

### 2.1 Generation of NE Organoids from Human iPSC in a Manipulatable Culture Environment

**Figure 1:**
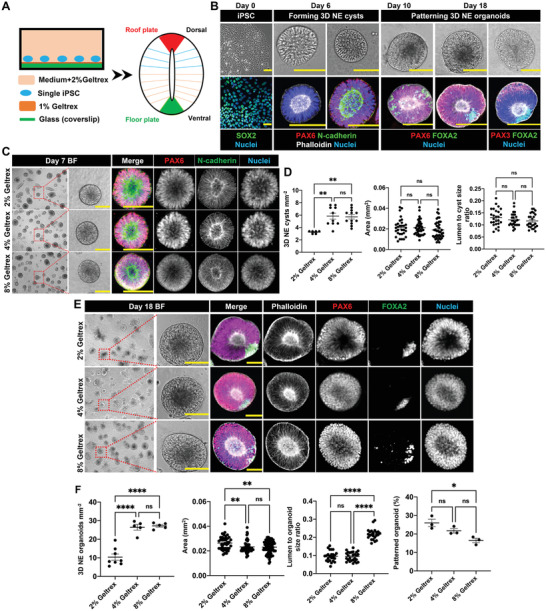
Formation of 3D NE organoids from human iPSCs. A) Culture system for generating 3D DV patterned NE organoids from single hiPSCs. B) Developmental phases during the formation of NE organoids from single hiPSCs. Phase 1, hiPSCs were digested into single cells and seeded on Geltrex‐coated glass coverslip on day 0. Phase 2, hiPSCs differentiated into NE cells and self‐organized into cystic structures with smooth outer surface and containing central lumen with apical‐basal polarity on day 6. Phase 3, development of NE cysts with FOXA2^+^ ventral NE cells and PAX3^+^ dorsal NE cells, forming DV patterned NE organoids. Top panels show bright‐field images of hiPSCs, NE cysts, and NE organoids. Bottom panels show immunofluorescence staining of pluripotency marker SOX2, F‐actin (phalloidin), N‐cadherin, early neuroectodermal marker PAX6, dorsal marker PAX3, and ventral marker FOXA2 in corresponding phases during NE organoid formation. Scale bars: 100 µm. C) Representative bright‐field (BF) images of NE cysts on day 7 and the corresponding immunofluorescence staining of PAX6, N‐cadherin, and nuclei in NE cysts formed in the presence of 2%, 4%, and 8% Geltrex in the neural induction medium. Scale bars: 100 µm. D) Quantification of the number density, area, and lumen‐to‐cyst size ratio of NE cysts on day 7, *n* ≥ 5. E) Representative bright‐field (BF) images (scale bars: 100 µm) and the corresponding immunofluorescence staining (scale bars: 50 µm) of F‐actin (phalloidin), PAX6, FOXA2, and nuclei of NE organoids on day 18 formed in the presence of 2%, 4%, and 8% Geltrex in the neural induction medium. F) Quantification of the number density, area, lumen‐to‐organoid size ratio, and the percentage of floor‐plate patterned NE organoids on day 18, *n* ≥ 3. Error bars in (D) and (F) represent S.E.M. *P*‐values of statistical significance were represented as: * *P* < 0.05, ** *P* < 0.01, *** *P* < 0.001, **** *P* < 0.0001, ns (not significant) *P* > 0.05. Using one‐way analysis of variance (ANOVA) followed by a Tukey's multiple comparisons test.

To enable investigation into the relative roles of intracellular and extracellular cues in NE organoids, an important first step is to develop a 3D organoid culture system that allows simple, one‐pot manipulations of these cues. Previous studies have shown successful production of DV patterned NE organoids in a 3D culture system by embedding pluripotent stem cells in hydrogels or growing them on hydrogel beds.^[^ ^4^ ^]^ Inspired by this approach, we seeded single hiPSCs at 8000–10 000 cells cm^−2^ on 1% (v/v) Geltrex‐coated glass surface and further added 2% (v/v) Geltrex to the neural induction medium to provide a biomimetic environment for the 3D development of NE organoids (**Figure** **1**A). Under this condition, single hiPSCs successfully self‐aggregated into 3D cystic structures with smooth outer surface and containing central lumen with apical–basal polarity on day 6, as evidenced by the apical enrichment of N‐cadherin (Figure 1B). Concurrently, the hiPSCs differentiated into neural epithelial cells, indicated by the expression of early neuroectodermal marker PAX6 (Figure 1B). After addition of AtRA and Shh from day 4 to day 9, FOXA2^+^ ventral NE cells started to be present in the NE cysts on day 10 and continued to accumulate in the floor‐plate region. At the same time, PAX3^+^ dorsal NE cells became detectable in the opposite roof‐plate region, displaying key architectural features of a DV patterned NE organoid with lumen structure on day 18 (Figure 1B). These data show that our simple culture system can be used for producing NE organoids from single hiPSCs, which undergo distinct developmental phases of the formation of luminal NE cysts and DV patterning of the NE organoids. Moreover, the culture configuration enables both microscopic monitoring and longitudinal manipulation of the organoid formation process through cellular and extracellular cues.

The out‐of‐plane morphogenesis in our approach is facilitated by the presence of Geltrex in the medium. We therefore asked whether varying Geltrex concentration in the neural induction medium can affect the self‐organization of NE cysts and organoids. This is particularly interesting in light of a recent study that showed that higher arginine–glycine–aspartate (RGD) ligand density in hiPSC culture results in a higher rate of lumen formation.^[^ ^18^ ^]^ We found that, under different Geltrex concentrations in the medium (2%, 4%, and 8% v/v), single hiPSCs were able to self‐assemble and differentiate into NE cysts (Figure 1C) and DV patterned NE organoids (Figure 1E; Figure S1A,B, Supporting Information). These 3D multicellular structures kept growing and increased in size throughout the complete culture process from day 0 to day 18, with comparable developmental progression (Figure S1C, Supporting Information). Interestingly, increasing Geltrex concentration led to a non‐monotonic effect in cellular self‐organization. At the initial phase of NE cyst formation, raising Geltrex concentration from 2% to 4% nearly doubled the number of formed 3D NE cysts, without affecting cyst or lumen size (Figure 1D). However, a further increase to 8% Geltrex concentration did not translate to a further increase in the rate of NE cyst formation (Figure 1D). These effects carried forward to the later DV patterning stage. Remarkably, 4% and 8% Geltrex both decreased the NE organoid size (Figure 1F). Moreover, 8% Geltrex resulted in significantly larger lumen‐to‐organoid size ratio and lower patterning success of the NE organoids (Figure 1F). Altogether, our data indicate for the first time that the formation and morphological features of NE organoids can be manipulated through modulation of the 3D culture condition, by changing the concentration of soluble Geltrex in the medium.

### 2.2 Actomyosin Contractility Regulates NE Cyst Formation and Floor‐Plate Patterning

**Figure 2:**
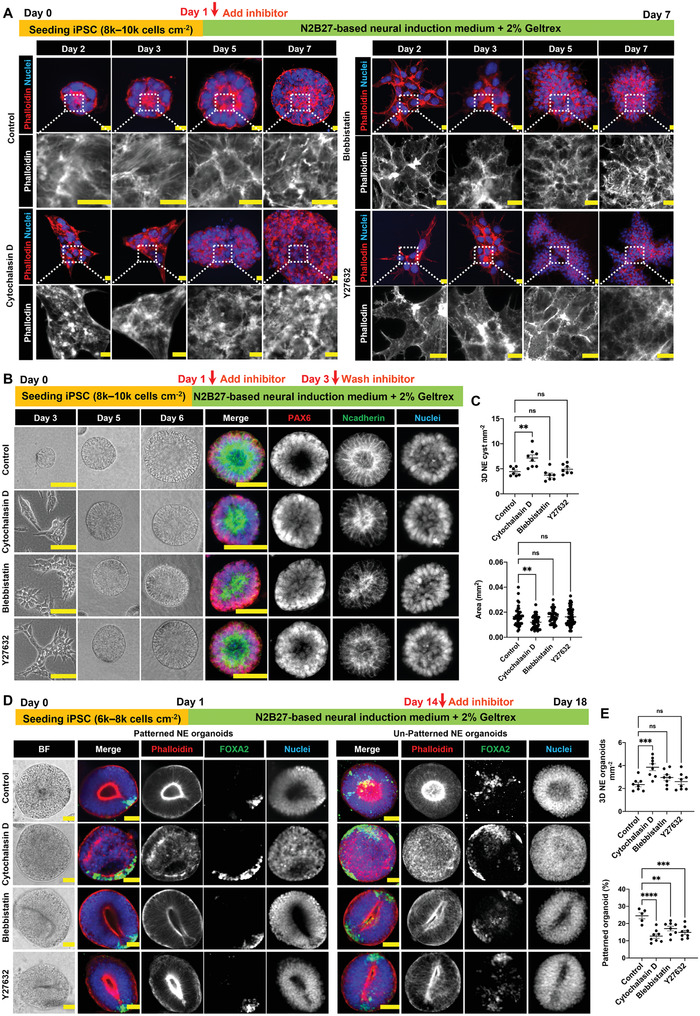
Actomyosin contractility is crucial for 3D NE organoid construction and patterning. A) Immunofluorescence staining of F‐actin (phalloidin) in NE cysts from day 2 to day 7 during NE cyst formation. Y276329 (10 × 10^−6^ m), (±)‐Blebbistatin (10 × 10^−6^ m), or cytochalasin D (0.2 × 10^−6^ m) was added from day 1 to day 7. Scale bars: 10 µm. B) Bright‐field images of NE cysts (scale bars: 100 µm) and the corresponding immunofluorescence staining of N‐cadherin and PAX6 in NE cysts (scale bars: 50 µm) on day 7. Inhibitors were added on day 1 and washed out on day 3. C) Quantification of number density and area of NE cysts on day 7 after inhibitor washout on day 3, *n* ≥ 7. D. Bright‐field images of NE organoids and the corresponding immunofluorescence staining of F‐actin (phalloidin) and FOXA2 in NE organoids on day 18, showing representative examples of successful and unsuccessful floor plate patterning in the organoids. Inhibitors were added from day 14 to day 18 during NE organoid culture. Scale bars: 50 µm. E) Quantification of number density of NE organoids and the percentage of floor plate patterned NE organoids on day 18 under different treatments, *n* ≥ 5. Error bars in (C) and (E) represent S.E.M. *P*‐values of statistical significance were represented as: * *P* < 0.05, ** *P* < 0.01, *** *P* < 0.001, **** *P* < 0.0001, ns (not significant) *P* > 0.05, using one‐way analysis of variance (ANOVA) followed by Dunnett multiple comparisons test.

It is generally accepted that the actin cytoskeleton is crucial for morphogenetic processes,^[^ ^11^, ^13^, ^19^ ^]^ and actin filaments have been shown to be mechanically responsive to external forces during the formation of well‐organized NE organoid structure.^[^ ^6^, ^7^ ^]^ However, the active role of actomyosin contractility in the different cellular differentiation and self‐organization phases during NE organoid formation is still elusive. To investigate this, we performed a systematic inhibition study, exploiting the possibility of probing our organoids at different developmental phases. At the NE cyst formation phase, single hiPSCs gradually differentiated, self‐aggregated, and formed polarized lumen structure after day 3, accompanied by the clear presence of distinct actin stress fibers throughout the process until day 7 (**Figure** **2**A; Figure S2, Supporting Information). Inhibiting actin polymerization by addition of cytochalasin D on day 1 resulted in fragmented actin structures in the cells, while inhibition of nonmuscle myosin II by blebbistatin as well as upstream Rho‐associated kinase (ROCK) by Y27632 prevented formation of actin stress fibers (Figure 2A). In all cases with the inhibitor treatments, the individual hiPSCs failed to self‐aggregate into 3D cysts with lumen structure, despite positive PAX6 expression (Figure 2A; Figure S3A, Supporting Information). Subsequent washout of inhibitors on day 3 allowed the cells to reaggregate into 3D cysts structure with polarized lumen (Figure 2B; Figure S3B, Supporting Information). Within 3 days after inhibitor washout, no statistically significant difference in the area and number density of 3D NE cysts was found between control, blebbistatin‐treated, and Y27632‐treated samples. By contrast, the cytochalasin D‐treated samples presented higher number density but smaller NE cysts (Figure 2C), and with less well‐defined luminal surface (Figure S3B, Supporting Information). These results indicate that active actin dynamics are required for the morphogenetic processes behind the 3D formation of luminal NE cysts. To test whether actin dynamics are also required for the maintenance of the 3D cellular architecture, we applied the inhibitors on day 5, after the successful initial formation of the 3D luminal cysts. Indeed, all inhibitors caused collapse of the polarized lumen in 3D NE cysts (Figure S4A, Supporting Information) and disassembly of the already formed cysts, resulting in significantly sparser NE cysts (Figure S4B, Supporting Information).

We next asked whether actomyosin contractility is equally critical in the later phase of NE organoids formation and floor‐plate patterning. When the inhibitors were added on day 14, bright‐field imaging and immunofluorescence staining showed that NE organoids in the inhibitor‐treated samples maintained an overall morphology comparable to the control samples on day 18 (Figure 2D). However, blebbistatin and Y27632 treatments led to narrow, elongated lumen (Figure 2D) and significantly reduced the success rate in floor‐plate patterning compared to the control samples (Figure 2E). Interestingly, cytochalasin D‐treated samples exhibited increased number density of NE organoids compared to control group (Figure 2E), but 100% of the organoids lost their lumen and the percentage of floor‐plate patterned organoids dropped to half of the percentage in the control samples (Figure 2D,E). Together, these data demonstrate the importance of actin structures in both the NE cyst formation phase and the floor‐plate patterning phase of NE organoids.

### 2.3 YAP Is Involved in NE Cyst Formation but not Floor‐Plate Patterning

**Figure 3:**
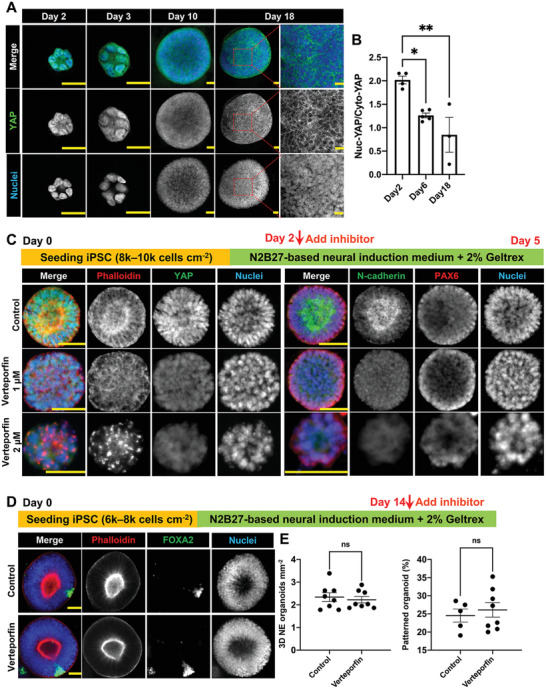
The dynamic role of YAP in the NE cyst formation and NE organoid patterning. A) Immunofluorescence staining of YAP in NE organoids from day 2 to day 18 during NE organoid formation. Scale bars: 25 µm. B) Quantification of nuclear‐to‐cytoplasmic YAP intensity ratio on day 2, day 6, and day 18, *n* ≥ 3. C) Immunofluorescence staining of F‐actin (phalloidin), YAP, N‐cadherin, and PAX6 in NE cysts on day 5. Verteporfin (0, 1, or 2 × 10^−6^ m) was added from day 2 to day 5 during NE cyst culture. Scale bars: 50 µm. D) Immunofluorescence staining of F‐actin (phalloidin) and FOXA2 in NE organoids on day 18. Verteporfin (1 × 10^−6^ m) was added from day 14 to day 18 during 3D NE organoid culture. Scale bars: 50 µm. E) Quantification of number density of NE organoids and the percentage of floor plate patterned NE organoids on day 18 under different treatments, *n* ≥ 5. Error bars in (B) and (E) represent S.E.M. *P*‐values of statistical significance were represented as: * *P* < 0.05, ** *P* < 0.01, ns (not significant) *P* > 0.05, using one‐way analysis of variance (ANOVA) followed by Dunnett multiple comparisons test (B) and Student's *t*‐test (E).

The transcriptional regulator YAP is known to mediate the ability of cells to sense and respond to physical cues from the environment, by shuttling between the nucleus (therefore functionally active) and the cytoplasm (inactive state).^[^ ^20^, ^21^, ^22^, ^23^ ^]^ Cellular processes involved in NE organoid formation, such as cell contractility, substrate adhesion, and differentiation, have been found to be associated with YAP transcriptional activity.^[^ ^21^, ^22^ ^]^ Moreover, YAP has also recently been reported to regulate lumen formation in hiPSC‐derived cysts.^[^ ^18^ ^]^ Thus, we next assessed the role of YAP in the formation of luminal NE cysts and organoids. Temporal profiling of the YAP expression indicated that YAP localized mostly in the nuclei, where it is primed to be active, at the early stage (day 2–3), but thereafter translocated to the cytoplasm (**Figure** **3**A). Quantification of YAP intensity confirmed a gradual decrease of the nuclear‐to‐cytoplasmic YAP ratio from day 2 to day 18, where the ratio was close to 1 (1.17 ± 0.05) on day 6 (Figure 3B). This suggests that YAP is involved in the regulation of luminal NE cysts. To test this, we added specific YAP inhibitor, verteporfin, in the early phase of NE cyst formation. Adding verteporfin at a low concentration (1 × 10^−6^ m) on day 1 (at the stage of single hiPSC culture) repressed YAP expression in the cells and led to dramatic consequences, such as disruption of actin structure, failure of the initiation of lumen formation, and sometimes loss of nuclear integrity in 3D NE cysts on day 2 and 3 (Figure S5, Supporting Information). To specifically target the lumen polarization phase, which was consistently observed on day 4, we added verteporfin in the culture medium from day 2 until day 5. Addition of 1 × 10^−6^ m verteporfin suppressed YAP expression without affecting nuclear integrity nor the overall cystic structure, but abrogated lumen formation and polarization in the NE cysts (Figure 3C). Increasing verteporfin concentration to 2 × 10^−6^ m resulted in more pronounced effects, with punctate actin aggregates and failure to form 3D cystic structure with smooth surface (Figure 3C). Given this importance of YAP in the initial formation of 3D luminal NE cysts, we then asked whether YAP also plays a role in the maintenance of cellular architecture and the subsequent floor‐plate patterning of NE organoids. When verteporfin was introduced from day 14 to day 18, no observable difference was found between the cellular organization, number density, and success rate of floor‐plate patterning of the NE organoids in the control and verteporfin‐treated samples (Figure 3D,E). This is consistent with the cytoplasmic localization and therefore lack of transcriptional activity of YAP in late‐stage NE organoids. Therefore, our data show that YAP is critical for lumen formation and polarity in NE cysts, but is not involved in regulating the patterning process of NE organoids.

### 2.4 Anisotropic Substrate Topographies Affect NE Cyst Formation and Floor‐Plate Patterning of NE Organoids

**Figure 4:**
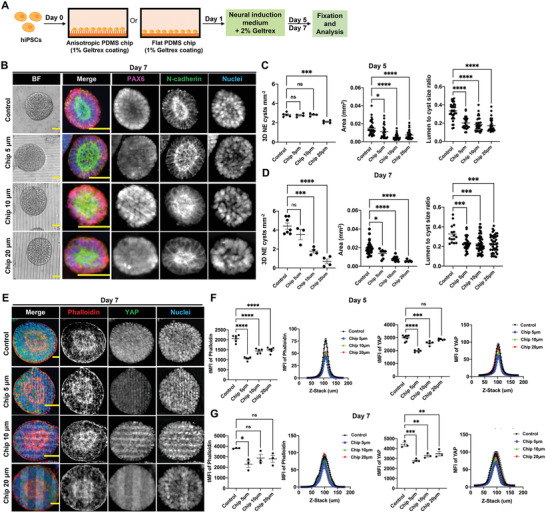
Anisotropic substrate topographies affect NE cyst formation. A) Schematic of growing hiPSC on PDMS chips with linear grooves and control flat PDMS chip to generate NE cysts. B) NE cysts growing on PDMS chip with linear grooves of different dimensions (Chip 5 µm, Chip 10 µm, Chip 20 µm) and control PDMS chip with flat surface. Bright‐field images and immunofluorescence staining of N‐cadherin and PAX6 in NE cysts on day 7. Scale bars: 50 µm. C,D) Quantification of number density, area, and lumen‐to‐cyst size ratio of NE cysts on (C) day 5 and (D) day 7, *n* ≥ 3. E) Immunofluorescence staining of F‐actin (phalloidin) and YAP in NE cysts growing on PDMS chip with linear grooves of different dimensions (Chip 5 µm, Chip 10 µm, Chip 20 µm) and control PDMS chip with flat surface on day 7. Scale bars: 20 µm. F,G) Quantification of F‐actin (phalloidin) and YAP intensity in NE cysts on (F) day 5 and (G) day 7, *n* ≥ 3. Total mean fluorescence intensity (tMFI) of F‐actin (phalloidin) and YAP were shown as dot plots and the corresponding MFI of each slide along the Z‐stack were indicated as curve graphs. Error bars in (C), (D), (F), and (G) represent S.E.M. *P*‐values of statistical significance were represented as: * *P* < 0.05, ** *P* < 0.01, *** *P* < 0.001, **** *P* < 0.0001, using one‐way analysis of variance (ANOVA) followed by Dunnett multiple comparisons test.

Cytoskeletal organization and YAP activity, which we have shown to be important for organoid formation and patterning, are known to be mechanosensitive and highly dependent on physical cues in the extracellular environment.^[^ ^11^, ^14^, ^15^, ^16^, ^17^ ^]^ Recent studies have also started to explore the exciting possibility of using external geometrical effects to influence the self‐organization process of 3D organoids.^[^ ^8^, ^9^ ^]^ Thus, we first sought to understand the impact of substrate topography in modulating NE cyst formation. polydimethylsiloxane (PDMS) chips were microfabricated with linear grooves of different dimensions using photolithography (see the Experimental Section). Specifically, the PDMS chips contain grooves and ridges with different lateral dimensions and spacings of 5 µm (Chip 5 µm), 10 µm (Chip 10 µm), and 20 µm (Chip 20 µm), respectively, and a height of 6 µm (Figure S6, Supporting Information). After being coated with a thin layer of 1% (v/v) Geltrex, the chips were used for growing hiPSCs (**Figure** **4**A). As shown by immunofluorescence staining, hiPSCs growing on these PDMS surface were able to self‐organize into NE cysts, which were defined with polarized lumen structure and which expressed neural epithelial cell marker PAX6 as well as apical N‐cadherin (Figure 4A,B). Compared to the number density of NE cysts obtained on control substrates (flat PDMS), lower NE cyst number density was observed on Chip 20 µm on day 5 (Figure 4C) and on both Chip 20 µm and Chip 10 µm on day 7 (Figure 4D). In addition, the size of the NE cysts and the lumen‐to‐cyst size ratio of the NE cysts derived from all anisotropic PDMS surfaces were significantly decreased on day 5 and day 7 compared to control (Figure 4C,D). Notably, we observed that hiPSCs were also able to self‐assemble along the orientation of anisotropic PDMS surface and form multicellular structures that exhibited polarized lumen structure but with jagged surface (Figure S7, Supporting Information). This suggests that the cells were able to sense and respond orientationally to the anisotropic substrate and that this topography sensing interferes with the NE cyst formation.

As topography sensing and the subsequent cellular contact guidance have been shown to be mediated by dynamic actomyosin,^[^ ^24^, ^25^, ^26^ ^]^ we hypothesized that the effects of the anisotropic substrate on NE cyst formation can be targeted by pretreating hiPSCs with ML7, a myosin light chain kinase inhibitor. Addition of ML7 (10 × 10^−6^ m) for the first 48 h indeed suppressed the formation of multicellular structures with anisotropic orientation and the formation of NE cysts were increased dramatically, interestingly for both anisotropic and flat (control) PDMS surfaces (Figure S8, Supporting Information). Consistent with this, the reduced NE cyst size and number density that were observed on anisotropic PDMS surfaces compared to on flat PDMS were largely erased in ML7 pretreated samples (Figure S8A,B, Supporting Information). Given the impact of anisotropic surface on actomyosin contractility and NE cysts formation, we also examined the actin organization and YAP expression in the NE cysts generated on different PDMS topographies. Total mean fluorescence intensity (tMFI) of both F‐actin (phalloidin) and YAP was significantly lower in NE cysts growing on all anisotropic PDMS surfaces on day 5, compared to on flat PDMS (Figure 4F). This decrease was still observed for YAP on day 7, but, interestingly, it diminished with time and significant difference in actin MFI was only detected between NE cysts on Chip 5 µm and on flat PDMS on day 7 (Figure 4E,G), suggesting that the 3D, out‐of‐plane growth of the NE cysts over time can attenuate the initial strong effect of substrate topography. Taken together, these results showed the capability of topographical features of the substrate in controlling NE cyst formation through modulation of cytoskeletal organization and YAP expression.

**Figure 5:**
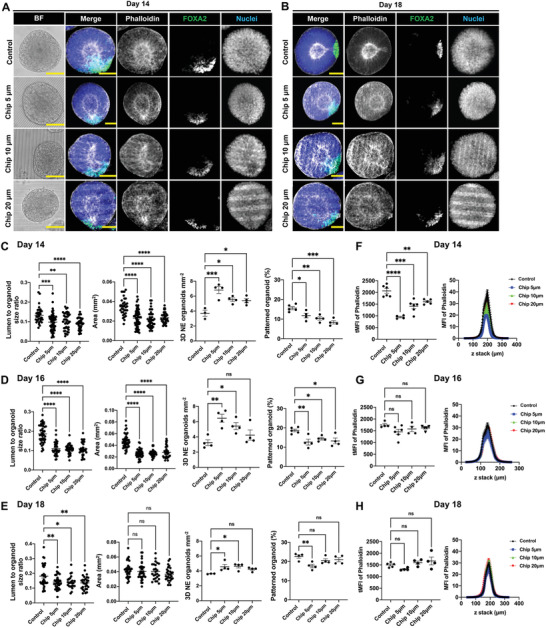
Anisotropic substrate topographies affect floor‐plate patterning of NE organoids. A,B) NE organoids growing on PDMS chip with linear grooves of different dimensions (Chip 5 µm, Chip 10 µm, Chip 20 µm) and control PDMS chip with flat surface. Bright‐field images (Scale bars: 100 µm) and immunofluorescence staining (Scale bars: 50 µm) of F‐actin (phalloidin) and FOXA2 in NE organoids on A) day 14 and B) day 18, showing representative examples of successful floor‐plate patterning in NE organoids. C–E) Quantification of area, lumen‐to‐organoid size ratio, number density of NE organoids and percentage of floor‐plate patterned NE organoids on (C) day 14, (D) day 16, and (E) day 18, n ≥ 3. F–H) Quantification of F‐actin (phalloidin) intensity in NE organoids on (F) day 14, (G) day 16, and (H) day 18, *n* ≥ 3. Total mean fluorescence intensity (MFI) of F‐actin (phalloidin) were shown as dot plots (left) and the corresponding MFI of each slide along the Z‐stack were indicated as curve graphs (right). Error bars in (C–H) represent S.E.M. *P*‐values of statistical significance were represented as: * *P* < 0.05, ** *P* < 0.01, *** *P* < 0.001, **** *P* < 0.0001, using one‐way analysis of variance (ANOVA) followed by Dunnett multiple comparisons test.

Next, we examined the late‐stage NE organoid formation on the PDMS topographies. NE organoids with smooth surface and well‐defined central lumen structures, with both successful and unsuccessful floor‐plate patterning, were observed on the PDMS substrates from day 14 (**Figure** **5**A,B; Figure S9, Supporting Information). Notably, compared to NE organoids grown on flat PDMS surface, higher number density of NE organoids was obtained on all anisotropic PDMS surfaces on day 14, but these organoids exhibited significantly lower lumen‐to‐organoid size ratio, smaller size, and decreased percentage of patterned organoids (Figure 5C). Strikingly, these topography dependences became progressively weaker with longer culture times (Figure 5D,E). Similarly, F‐actin (phalloidin) fluorescence intensity was significantly lower for NE organoids generated on the anisotropic topographies compared to on flat PDMS surface on day 14 (Figure 5F), but these differences were no longer detectable on day 16 and 18 (Figure 5G,H). These results indicate that substrate topographical cues can affect the early formation and patterning of NE organoids, but their relevance become increasingly diminished as the organoids mature.

### 2.5 3D Microniches Shape NE Cyst Formation and Affect Floor‐Plate Patterning of NE Organoids

**Figure 6:**
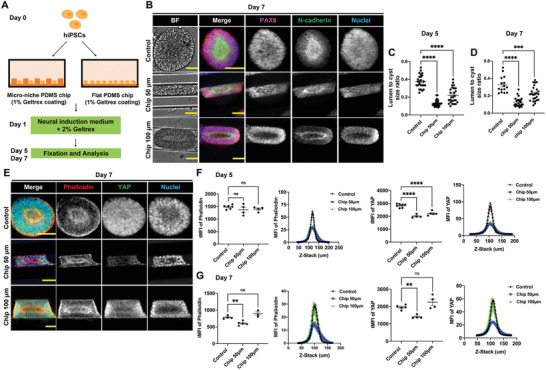
3D microniches guide NE cyst formation. A) Schematic of growing hiPSC on PDMS chips with linear grooves microniche and control flat PDMS chip to generate NE cysts. B) NE cysts growing on PDMS chip with linear grooves microniche of either 50 µm (Chip 50 µm) and 100 µm (Chip 100 µm) in width or 90 µm in height. Flat PDMS chip was used as control. Bright‐field images and immunofluorescence staining of N‐cadherin and PAX6 in NE cysts on day 7. Scale bars: 50 µm. C,D) Quantification lumen‐to‐cyst size ratio of NE cysts on (C) day 5 and (D) day 7, *n* ≥ 15. E) Immunofluorescence staining of F‐actin (phalloidin) and YAP in NE cysts growing on PDMS chip with linear grooves microniche of different dimensions (Chip 50 µm, Chip 100 µm) and control PDMS chip with flat surface on day 7. Scale bars: 50 µm. F,G) Quantification of F‐actin (phalloidin) and YAP intensity in NE cysts on (F) day 5 and (G) day 7, *n* ≥ 4. Total mean fluorescence intensity (MFI) of F‐actin (phalloidin) and YAP were shown as dot plots (left) and the corresponding MFI of each slide along the Z‐stack were indicated as curve graphs (right). Error bars in (C), (D), (F), and (G) represent S.E.M. *P*‐values of statistical significance were represented as: * *P* < 0.05, ** *P* < 0.01, *** *P* < 0.001, **** *P* < 0.0001, using one‐way analysis of variance (ANOVA) followed by Dunnett multiple comparisons test.

**Figure 7:**
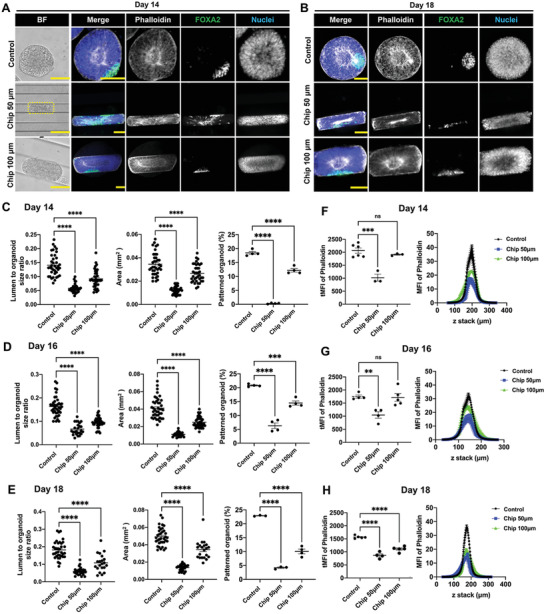
3D microniches affect floor‐plate patterning of NE organoids. A,B) NE organoids growing on PDMS chip with linear grooves microniche of different dimensions (Chip 50 µm, Chip 100 µm) and flat PDMS was used as a control. Bright‐field images (Scale bars: 100 µm) and immunofluorescence staining (Scale bars: 50 µm) of F‐actin (phalloidin) and FOXA2 in NE organoids on (A) day 14 and (B) day 18, showing representative examples of successful and unsuccessful floor‐plate patterning in NE organoids. C–E) Quantification of area, lumen‐to‐organoid size ratio of NE organoids and percentage of floor‐plate patterned NE organoids on (C) day 14, (D) day 16, and (E) day 18, n ≥ 3. F–H) Quantification of F‐actin (phalloidin) intensity in NE organoids on (F) day 14, (G) day 16, and (H) day 18, *n* ≥ 3. Total mean fluorescence intensity (MFI) of F‐actin (phalloidin) was shown as dot plots (left) and corresponding MFI of each slide along the Z‐stack were indicated as curve graphs (right). Error bars in (C–H) represent S.E.M. *P*‐values of statistical significance were represented as: * *P* < 0.05, ** *P* < 0.01, *** *P* < 0.001, **** *P* < 0.0001, using one‐way analysis of variance (ANOVA) followed by Dunnett multiple comparisons test.

Finally, we hypothesized that geometric confinement in the form of 3D anisotropic microniches could have an impact on shaping the self‐assembly of NE cysts and organoids from single hiPSCs. To test this hypothesis, we seeded single hiPSCs on PDMS chips with linear grooves of either 50 µm (Chip 50 µm) and 100 µm (Chip 100 µm) in width and 90 µm in height (Figure S6, Supporting Information; **Figure** **6**A), which were chosen to be sufficiently large to geometrically confine the growth of the organoids. As expected, the morphology of the multicellular structures self‐aggregated from hiPSCs followed the shape of these microniches (Figure 6B). Immunofluorescence staining showed that these multicellular structures expressed PAX6 and apical N‐cadherin and contained polarized central lumens, confirming a similar cystic structure as NE cysts growing on flat PDMS (Figure 6B). Despite this, the spatial confinement in the microniches resulted in significantly lower lumen‐to‐cyst size ratio on both day 5 and day 7 compared to those for the NE cysts formed on flat PDMS (Figure 6C,D). Interestingly, the effect is more pronounced with the 50 µm microniches than with the 100 µm microniches, highlighting the stronger confinement effect in the former. This is furthermore mirrored by a similar trend in the intracellular regulation of actin and YAP (Figure 6E–G), where both total MFI of F‐actin (phalloidin) and YAP were found to be significantly lower in NE cysts from the 50 µm microniches, but not the 100 µm microniches, compared to on flat PDMS after 7 days of culture (Figure 6G). To further examine the effect of microniches on floor‐plate patterning of NE organoids, the NE cysts were kept growing on the PDMS chips. The morphology of the NE organoids maintained similar shape as 3D microniches and both successful and unsuccessful floor‐plate patterning were observed in the luminal NE organoids (**Figure** **7**A,B; Figure S10, Supporting Information). However, NE organoids growing in the 50 and 100 µm microniches were generally characterized with lower lumen‐to‐organoid size ratio, smaller size, and reduced expression of actin from day 14 to day 18 (Figure 7C–H). Strikingly, all NE organoids in the 50 µm microniches failed to achieve floor‐plate patterning on day 14 and the percentage of patterned NE organoids in these microniches was generally very low compared to in other conditions (Figure 7C–E). These results suggest that geometrical confinement can have a strong effect on the late‐stage patterning phase of NE organoids.

## 3 Discussion

**Figure 8:**
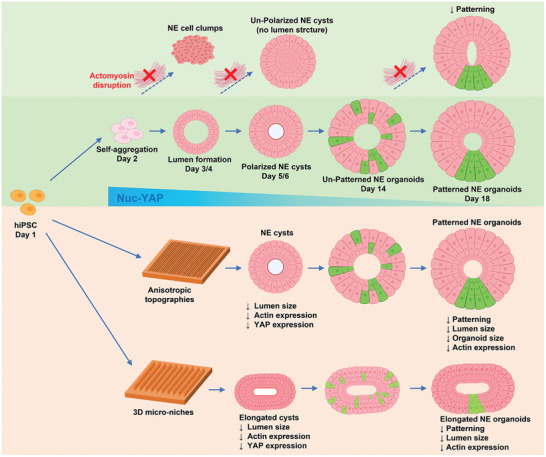
Schematic illustration of the interfacial and intracellular regulation of NE organoid formation and development. Single hiPSCs self‐organize into floor‐plate patterned NE organoids under neural induction condition during culture of 18 days, during which the role of YAP gradually decreases (middle). Disruption of the cytoskeletal organization and actomyosin contractility severely impacts different stages in the process, resulting in failure to form cysts, lumen structures, and floor‐plate patterning (top). Furthermore, different interfacial cues presented on the substrate modulate the cellular self‐assembly process, the organoid morphology, and the cell organization within the organoids (bottom).

3D organoid models have emerged as unique in vitro models for studying organ development, function, and disease.^[^ ^27^, ^28^ ^]^ Conventional organoid generation relies on the encapsulation and growth of organoids in drops of solidified reconstituted extracellular matrices.^[^ ^29^, ^30^, ^31^, ^32^, ^33^ ^]^ Studying how stem cells sense different extracellular cues during organoid formation is technically challenging using this approach, as the organoids are fully embedded in a 3D microenvironment with local differences of both physical parameters and growth factor accessibility.^[^ ^31^, ^32^ ^]^ Recent studies have put forward alternative strategies of providing soluble extracellular matrix or phasing the reconstitution of the extracellular matrix to support 3D organoid formation, which can allow one to subject the cells to external physical cues during organoid formation.^[^ ^4^, ^34^, ^35^, ^36^ ^]^ In this work, we established a simple culture system to generate 3D NE organoids by growing hiPSCs on Geltrex‐precoated surface with soluble Geltrex in the medium, which furthermore enables easy manipulation of the biochemical and physical culture conditions of the organoids. Consistent with the features of both human and mouse NE organoids from previous studies,^[^ ^4^, ^5^, ^25^ ^]^ this system allows hiPSCs to self‐organize and differentiate into 3D spherical NE cysts with central lumen structure, which then develop into dorsal–ventral patterned NE organoids (**Figure** **8**). Note that there is an overall variation of NE organoid size and phenotype heterogeneity within each tested culture condition, although we did not observe any size‐dependent trend in NE organoid patterning. As we focused on the floor‐plate patterning process, which was detectable after 18 days of culture in this system, we have not gone for periods of culture longer than 18 days to further follow the growth of the NE organoids in size. Our strategy of providing both Geltrex substrate coating and soluble Geltrex in the medium to support 3D growth appears to circumvent a previous observation that hPSCs growing on Geltrex precoated surface self‐assembled into multicellular structures with jagged outside surface, whereas NE organoids with smooth surface were obtained only on Geltrex bed, which was attributed to differences in the matrix rigidity.^[^ ^4^ ^]^ At the same time, these setups present different ligand density, availability, and distribution to the cells, and a recent study indeed demonstrated that higher RGD density, but not matrix stiffness, is associated with lumen formation in hiPSC clusters.^[^ ^18^ ^]^ Consistent with this, our results indicate that increasing soluble Geltrex concentration in the medium leads to higher lumen‐to‐organoid size ratio. Moreover, we show that higher percentage of soluble Geltrex promotes the formation of NE cysts and NE organoids on Geltrex precoated surfaces under neural induction environment, providing a simple experimental handle to control NE organoid formation.

NE organoid morphogenesis includes apical–basal polarity of lumen and cell‐fate determination of DV patterning. Previous studies have demonstrated that cytoskeletal organization is crucial for constructing NE organoids from single hiPSC under neural induction conditions.^[^ ^7^ ^]^ However, the role of cytoskeletal remodeling in the different phases of NE organoids morphogenesis was still unclear. Our study reveals that, during the initiation phase from single hiPSCs, actin polymerization inhibition using cytochalasin D, nonmuscle myosin II inhibition by blebbistatin, as well as ROCK inhibition by Y27632 all prevented single hiPSCs self‐aggregate into 3D cystic structures. This effect is reversible, as washout of these inhibitions at an early stage enables reconstruction of well‐organized 3D cystic structures from cell clusters. By contrast, during the later stage of NE cysts formation, inhibition of F‐actin filament formation and polymerization causes loss of apical–basal polarity or even the complete lumen structure. Moreover, during the late‐stage floor‐plate patterning, these inhibitions significantly repressed the cellular patterning but led to distinct effects on the lumen architecture of the NE organoids. ROCK and myosin II inhibition resulted in NE organoids with elongated lumen, whereas under cytochalasin D treatment the NE organoids completely lost the lumen structure, suggesting that NE organoids may have a contractility‐independent compensatory mechanism for the maintenance of lumen. These results illustrate a dynamic function of cytoskeleton organization in controlling cellular self‐aggregation, lumen formation and polarization, as well as floor‐plate patterning during different stages of NE organoid formation (Figure 8). Recent study with hiPSC‐derived cystic structures also described the involvement of the transcriptional regulator YAP in lumen formation.^[^ ^18^ ^]^ It was shown that, in hiPSC clusters, YAP is initially localized in the nucleus, indicative of YAP being active, and as the lumens form over time, YAP translocates to the cytoplasm, where it is inactive.^[^ ^18^ ^]^ In line with this observation, we found that, under neural induction conditions, YAP is also localized in the nucleus at the early stage of NE cyst formation and gradually translocates to the cytoplasm toward the end of NE organoids formation (Figure 8). Furthermore, our YAP inhibition studies at different developmental stages reveals that YAP is especially crucial for maintaining apical–basal polarity of lumen during NE cyst formation stage.

Previous studies on 2D substrates by our group and others have demonstrated that cytoskeletal organization and YAP activity can be sensitively tuned by biophysical cues in the cellular microenvironments, such as topography, geometry, and confinement.^[^ ^14^, ^37^, ^38^, ^39^, ^40^ ^]^ However, the role of these biophysical cues on in the context of 3D cellular self‐organization and organoids has only started to be explored.^[^ ^8^, ^9^, ^10^ ^]^ For instance, a recent study presents a chip‐based culture system to control the patterning and morphogenesis of intestinal organoids via the physical properties.^[^ ^10^ ^]^ In our study, when hiPSCs were seeded on anisotropic surface, the self‐aggregated multicellular morphology develops along the orientation of linear grooves, indicating that cells are able to sense and respond orientationally to the anisotropic substrate. This is followed by decreased formation density, area, and lumen‐to‐cyst size ratio of the NE cysts growing on anisotropic surface. This impact of interfacial anisotropy on NE cysts further extends to the NE formation, as the lumen‐to‐organoid size ratio, organoid area, and percentage of patterned organoid are significantly reduced (Figure 8). When the surface geometrical cues are large enough to confine the NE organoids in microniches, the NE cysts and organoids are micropatterned with similar morphology as the 3D microniches, exhibiting decreased lumen‐to‐cyst size ratio (for NE cysts) and smaller lumen‐to‐organoid size ratio (for NE organoids), as well as lower percentage of patterned organoids (Figure 8). These observations indicate that both approaches can strongly influence NE cyst and organoid formation process. Future studies can further explore the exciting possibility to manipulate NE organoid self‐organization, such as controlling the localization of floor‐plate formation, by systematically examining the 3D spatial distribution of cell phenotype in a large number of patterned NE organoids. Temporal quantification of actin and YAP expressions indicate a repression of actin filaments formation and YAP activity in NE cysts forming on anisotropic substrates and in 3D microniches. Moreover, decreased actin filaments formation is also detected in NE organoids induced by geometrical cues. Thus, cytoskeleton and YAP in NE cysts and organoids maintain mechanosensitive capability to response to different extracellular interfacial cues. In other words, our study provides evidence that external interfacial cues can potentially be exploited to modulate or even steer NE organoid development through mechanosensitive intracellular regulation. Consistent with this idea, previous studies of growing NE organoids in a poly(ethylene glycol) hydrogel‐supported 3D culture system have also started to explore how matrix rigidity can affect NE organoid formation and patterning.^[^ ^6^, ^7^ ^]^ In addition, our results also show varying Geltrex concentration in the neural induction medium can affect the self‐organization of NE organoids, indicating the potential effect of extracellular biochemical signal on shaping NE organoid formation.

The mechanosensitive regulation in NE organoids conferred by interfacial cues appears to be both space‐ and time‐dependent. For instance, the dimensions of the anisotropic linear grooves, the size of the 3D microniches, as well as the growing timepoint of NE organoids can all influence the interfacial geometric effects. In addition, the downregulation of YAP in NE cysts on the groove topographies starts later (from day 7) than in the 3D microniches (from day 5). On other hand, the downregulation of actin on the groove topographies starts earlier (from day 5 for NE cysts and from day 14 for NE organoids) than in the 3D microniches (from day 7 for NE cysts and from day 18 for NE organoids). These data suggest that actin cytoskeleton and YAP may be involved through different mechanisms in the sensing of interfacial cues in NE cysts and NE organoids. Particularly, not only the dynamic of actin cytoskeleton, but other different modulators are able to independently regulate YAP activity triggered by extracellular biophysical cues.^[^ ^21^, ^22^ ^]^ Coupled with the differential roles of actin and YAP in regulating NE organoid formation at different developmental stages, the response of actin and YAP to different extracellular geometry suggests their dynamic interplay in the cellular self‐organization process of NE organoids occurring on anisotropic substrates and 3D microniche, which should be further explored. Given the mechanoresponsiveness of both F‐actin and YAP to interfacial geometrical cues, we speculate that NE organoid formation and patterning can also be modulated by other mechanical factors via these pathways. In line with this hypothesis for NE organoids, a recent study on intestinal organoids similarly suggests that geometrical cues can affect intestinal organoids through a mechanoresponsive machinery.^[^ ^10^ ^]^ Altogether, our study demonstrates that modulation of the self‐organization process of NE organoids could be achieved through interfacial cues. Understanding how such cues in the niche regulate stem cell function and fate determination contributes to a better understanding of how cells develop distinctive morphologies, providing guidance for the design of 3D organoid generation system.

## 4 Conclusion

The result presented in this study highlight the importance of both intracellular regulation and interfacial cues in modulating NE organoid formation at different stages. A better understanding of how interfacial cues determine cell fate and tissue spatial organization in 3D organoids will pave the way for next‐generation methods for establishing and engineering organoids with higher complexity in terms of both cellular composition and tissue architecture.

## 5 Experimental Section

### Reagents and Antibodies

The following reagents were purchased and used: mTeSR1 medium (Stem Cell Tech, #5850), ReLeSR (Stem Cell Tech, #5872), DMEM/F12 medium (Invitrogen, #11330‐032), neurobasal medium (Invitrogen, #21103‐049), advanced DMEM‐F12 (Thermo Fisher, #12634010), N2 supplement 50× (Thermo Fisher, #17502001), B27 supplement 100× (Invitrogen, #17504044), MEM non‐essential amino acids (MEM‐NEAA) 100× (Thermo Fisher, #11140‐050); l‐glutamine (Gibco, #25030149), Geltrex LDEV‐free, hESC‐qualified, RGF‐BM matrix (Thermo Fisher, #A1413302); *β*‐mercaptoethanol (Sigma‐Aldrich, #M3148); accutase (Sigma‐Aldrich, #A6964‐500ML); SB431542 (Abcam, #ab120163), LDN193189 dihydrochloride (Sigma‐Aldrich, SML0559‐5MG), Y27632 (C_14_H_21_N_3_O·2HCl) (Stem Cell Tech, # 72304), all‐trans retinoic acid (AtRA) (Stem Cell Tech, #72262), recombinant human sonic hedgehog (Shh) (Peprotech, #100‐45), (±)‐Blebbistatin (Sigma‐Aldrich, #203390‐5MG), cytochalasin D (Sigma‐Aldrich, #C8273‐1MG), ML7 (Sigma‐Aldrich, # I2764‐5MG), Verteporfin (Sigma‐Aldrich, # SML0534‐5MG), paraformaldehyde, 16% w/v (Fisher Scientific, #11400580); Triton X‐100 (Sigma‐Aldrich, # 086031000), Sylgard 184 Silicone Elastomer Kit (Dow Corning, # 1317318), phosphate‐buffered saline (PBS) (Sigma‐Aldrich, #P4417), Hoechst 33342 (Invitrogen, #H3570), Alexa Fluor 647 phalloidin, 1/300 dilution (Thermo Fisher, #A22287), Alexa Fluor 488 phalloidin, 1/300 dilution (Thermo Fisher, # A12379). The following primary antibodies were used: YAP antibody, mouse monoclonal IgG2a, 1/80dilution (Santa Cruz, # SC101199); PAX6 antibody, polyclonal Mouse IgG, 1/300 dilution (Invitrogen, # MA1‐109); Ncadherin (CD325) antibody, polyclonal rabbit IgG, 1/300 dilution (Abcam, #ab18203); FOXA2 antibody, monoclonal Mouse IgG2a (1C7),1/200 dilution (Sigma‐Aldrich #SAB1403929); FOXA2 antibody, monoclonal Rabbit IgG (D56D6), 1/400 dilution (Cell Signaling Tech #8186); PAX3 antibody, polyclonal Rabbit IgG, 1/250 dilution (Invitrogen #38‐1801). The following secondary antibodies were used: Alexa Fluor 488 conjugated donkey antirabbit IgG (H+L),1/250 dilution, (Invitrogen, #A‐21206); Alexa Fluor 555 conjugated donkey antirabbit IgG (H+L), 1/250 dilution, (Invitrogen, #A‐31572); Alexa Fluor 647 conjugated donkey anti‐rabbit IgG (H+L), 1/250 dilution, (Invitrogen, #A‐31573); Alexa Fluor 488 conjugated donkey antimouse IgG (H+L),1/250 dilution, (Invitrogen, # A‐21202); Alexa Fluor 647 conjugated donkey antimouse IgG (H+L), 1/250 dilution (Invitrogen, # A‐31571).

### Human iPSC Culture

hiPSCs were generated and cultured as described previously.^[^ ^41^ ^]^ Briefly, hiPSCs were maintained in mTeSR1 medium and were cultured at 37 °C in 5% CO_2_ humidified incubator. Geltrex diluted in DMEM/F12 medium with a v/v ratio of 1:90 was used for coating cell culture flasks at 37 °C for at least 45 min before seeding hiPSCs. Medium was changed daily and cells were passaged every 4–6 days either by using ReLeSR or Accutase, and 10 × 10^−6^ m ROCK inhibitor Y27632 was added to the medium for the first 24 h during passaging.

### 3D Neural Epithelial Organoid Culture

Generation of 3D neural epithelial organoid were performed following previous protocol.^[^ ^4^ ^]^ Briefly, after hiPSC colonies were treated with Accutase for 4–6 min at 37 °C, single hiPSC cell suspension was prepared in mTeSR1 medium with Y27632 (10 × 10^−6^ m). hiPSCs at an initial cell density of 8000–10 000 cells cm^−2^ were plated on substrate with 1% Geltrex coating and cultured overnight. For differentiating hiPSC to neural epithelial cells, medium was switched to neural induction medium (NIM) from the following day (day 1) and 3D NE cysts were maintained in NIM for 7 days. For inducing patterned NE organoids: NIM was also used from the following day (day 1) to day 4, subsequently culture medium was switched to patterning medium (PM) from day 4 to day 9, and then NIM was used from day 9 to day 18. The details of medium are described below, N2B27 medium: Advance DMEM/F12 (50% v/v), neurobasal medium (50% v/v), N2 (0.5×), B27 (0.5×), MEM‐NEAA (1×), l‐glutamine (2 × 10^−3^ m), and *β*‐mercaptoethanol (0.1 × 10^−3^ m). NIM: N2B27 medium was supplemented with SB431542 (10 × 10^−6^ m), LDN193189 (0.1 × 10^−6^ m), and 2% Geltrex (v/v). PM: SB431542 (10 × 10^−6^ m), LDN193189 (0.1 × 10^−6^ m), Shh (10 × 10^−9^ m), AtRA (1 × 10^−6^ m), and 2% Geltrex (v/v).

### PDMS Chip Fabrication and Characterization

PDMS chips containing micropatterned substrates were fabricated using photolithography as described previously.^[^ ^25^, ^42^ ^]^ Briefly, the features were produced on a silicon master wafer by deep reactive‐ion etching (Philips Innovation Services, Eindhoven, the Netherlands) from a chromium photomask (Toppan Photomask, Corbeil Essonnes, France). Solution of silicon elastomer (PDMS, Sylgard 184, Dow Corning) and curing agent with a weight ratio of 10:1 was mixed and degassed, which was subsequently poured onto the silicon master that was passivated using fluorosilane and cured in the oven for 20–30 min at 110 °C. Cured PDMS chips with desired features (Figure S6, Supporting Information) were then peeled off from the master, followed by cleaning with 70% ethanol and drying with compressed air, respectively. The chips were coated with 1% Geltrex before cell seeding. For verifying the dimension of PDMS chips after fabrication, cross‐sections of the PDMS chips were prepared using a sharp blade and images were captured using an optical microscope (Leica dmi8) with 10×/0.32 objective lens (Figure S6, Supporting Information).

### Immunofluorescence Staining

3D NE cysts and organoids were fixed with 4% paraformaldehyde at room temperature for 15–20 min and thereafter washed with PBS. For immunofluorescence staining, 3D cysts and organoids were incubated in blocking buffer (5% donkey serum in 0.3% Triton X‐100) for 60–90 min at room temperature. Subsequent primary antibodies were diluted in blocking buffer and used for staining at 4 °C overnight. After three times of PBS wash in the following day, secondary antibodies and/or phalloidin diluted in PBS were applied to 3D cysts and organoids for incubating 60 min at room temperature. Nuclei were stained and visualized by using Hoechst 33342 (1/2000 dilution) with incubation at room temperature for 20 min, followed by three PBS washes. Samples were mounted to glass slide with mounting solution and stored at 4 °C.

### Microscopy Imaging and Image Analysis

Bright‐field images were captured by using Leica DMi8 microscope (Leica) equipped with 10×/0.32 objective lens and immunofluorescent images were acquired in Leica TCS SP8X confocal microscope (Leica) equipped with 20×/0.75 objective lens. All acquired images were processed by Fiji.^[^ ^43^, ^44^ ^]^ Confocal images were taken with Z‐stacks and the distance between slices in each Z‐stack was the same (2 µm) for all samples. The number density of NE cysts and organoids was defined by counting the number of NE cysts and organoids per mm^2^; each dot shown in the graph represents a randomly chosen field of view where NE cysts or organoids was quantified. The size of NE cysts and organoids was quantified by measuring the projected surface area of NE cysts and organoids; each dot shown in the graph represents one measured NE cyst or organoid. Lumen‐to‐cyst size ratio or lumen‐to‐organoid size ratio was calculated by dividing the projected surface area of the lumen by the projected surface area of the corresponding NE cyst or organoid, respectively; each dot shown in the graph represents one measured NE cyst or organoid. For quantitative analysis of the fluorescence intensity, images were taken by confocal microscope with identical setting between different samples. MFI of each slide along the Z‐stack of a 3D cyst or organoid sample was profiled in Fiji.^[^ ^43^, ^44^ ^]^ tMFI was calculated by adding the MFI value from all slices of a corresponding cyst or organoid sample together; each dot shown in the graph represents one measured NE cyst or organoid. For quantifying the YAP fluorescence intensity ratio between cytoplasm and nuclei, the Z‐stacks were first deconvolved through express deconvolution in Huygens professional (Scientific Volume Imaging B.V.) for segmentation of YAP fluorescence signal from the cytoplasm and nuclei, and then the YAP fluorescence intensity ratio was calculated by dividing the MFI of YAP from the cytoplasm to that from the nuclei. The percentage of patterned organoids was calculated by dividing the number of floor‐plate patterned organoids by the number of total organoids from the same randomly chosen field of view; each dot shown in the graphs represents a randomly chosen field of view where the percentage of patterned organoids was quantified.

### Statistical Analysis

All statistical analyses were conducted using Graphpad Prism 8. A Student's *t*‐test was performed to compare the statistical difference between two datasets. One‐way analysis of variance (ANOVA) followed by a Dunnett multiple comparisons test or Tukey's multiple comparisons test was performed when more than two datasets were compared. *P*‐values of statistical significance were represented as: * *P* < 0.05, ** *P* < 0.01, *** *P* < 0.001, **** *P* < 0.0001, ns (not significant) *P* > 0.05.

## Conflict of Interest

The authors declare no conflict of interest.

## Author Contributions

C.T. and N.A.K. conceived and designed the project. C.T. developed experimental approach, performed experiments, analyzed data, and prepared all figures. X.W. contributed to immunofluorescence staining experiments. M.D. helped with fluorescence intensity analysis. C.v.d.P. characterized the dimensions of the PDMS chips after fabrication. N.A.K. supported and supervised the project. C.T. and N.A.K. wrote the manuscript. All authors discussed and approved the manuscript.

## Supporting information

Supporting Information

## Data Availability

The data that support the findings of this study are available from the corresponding author upon reasonable request.
