# Supplementary material for: 3D Interfacial and Spatiotemporal Regulation of Human Neuroepithelial Organoids
Source: Adv Sci (Weinh). 2022 Jun 6;9(22):2201106. doi: 10.1002/advs.202201106 (PMC9353482; doi:10.1002/advs.202201106)
Supplement: Supplementary file 1 — Supporting Information [file ADVS-9-2201106-s001.pdf]

ADVANCED SCIENCE
Open Access

Supporting Information

for *Adv. Sci.*, DOI 10.1002/advs.202201106

3D Interfacial and Spatiotemporal Regulation of Human Neuroepithelial Organoids

*Chunling Tang, Xinhui Wang, Mirko D'Urso, Cas van der Putten and Nicholas A. Kurniawan**

Supporting Information

Three-dimensional interfacial and spatiotemporal regulation of human neuroepithelial organoids

*Chunling Tang, Xinhui Wang, Mirko D'Urso, Cas van der Putten, and Nicholas A. Kurniawan**

Dr. C. Tang, X. Wang, M. D'Urso, C. van der Putten, Dr. N.A. Kurniawan
Department of Biomedical Engineering, Eindhoven University of Technology, PO Box 513, 5600 MB Eindhoven, The Netherlands.
Institute for Complex Molecular Systems, PO Box 513, 5600 MB Eindhoven, The Netherlands.
E-mail: n.a.kurniawan@tue.nl

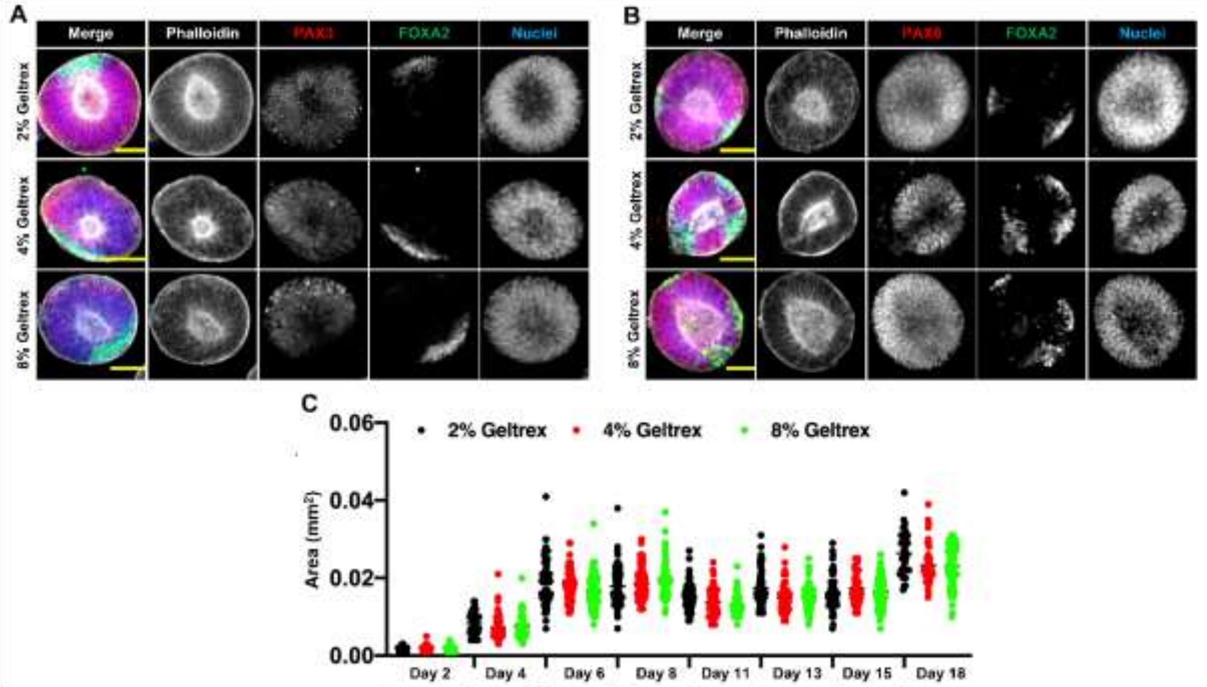

Figure S1. Growth and DV patterning of NE organoids. A and B. Examples of (A) successful and (B) unsuccessful DV patterning of NE organoids generated under different culture conditions with 2%, 4%, and 8% Geltrex in the neural induction medium. Immunofluorescence staining of F-actin (phalloidin), PAX3 (or PAX6), and FOXA2 in NE organoids on day 18. Scale bars: 50 μm. C. Growth curve of NE organoids. Area quantification of NE organoids growing in different culture conditions with 2%, 4%, and 8% Geltrex in neural induction medium from day 2 to day 18, $n \geq 5$. Each dot represents one organoid sample and at least 30 samples were quantified for each group at each timepoint.

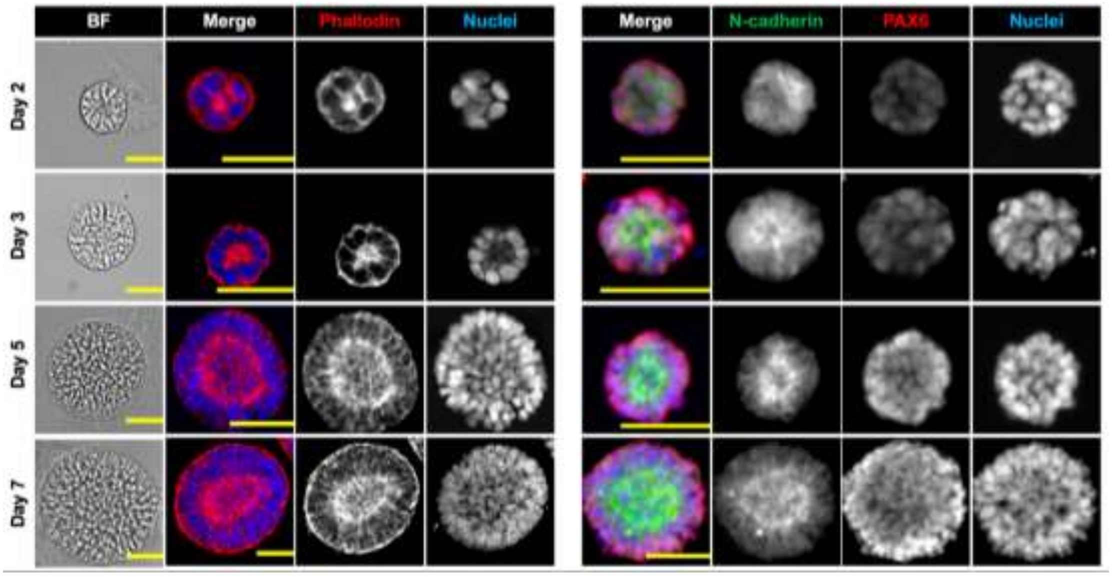

Figure S2. Bright-field images of NE cysts and immunofluorescence staining of F-actin (phalloidin), N-cadherin, and PAX6 in NE cysts from day 2 to day 7 during NE cyst formation. Scale bars: 50 µm

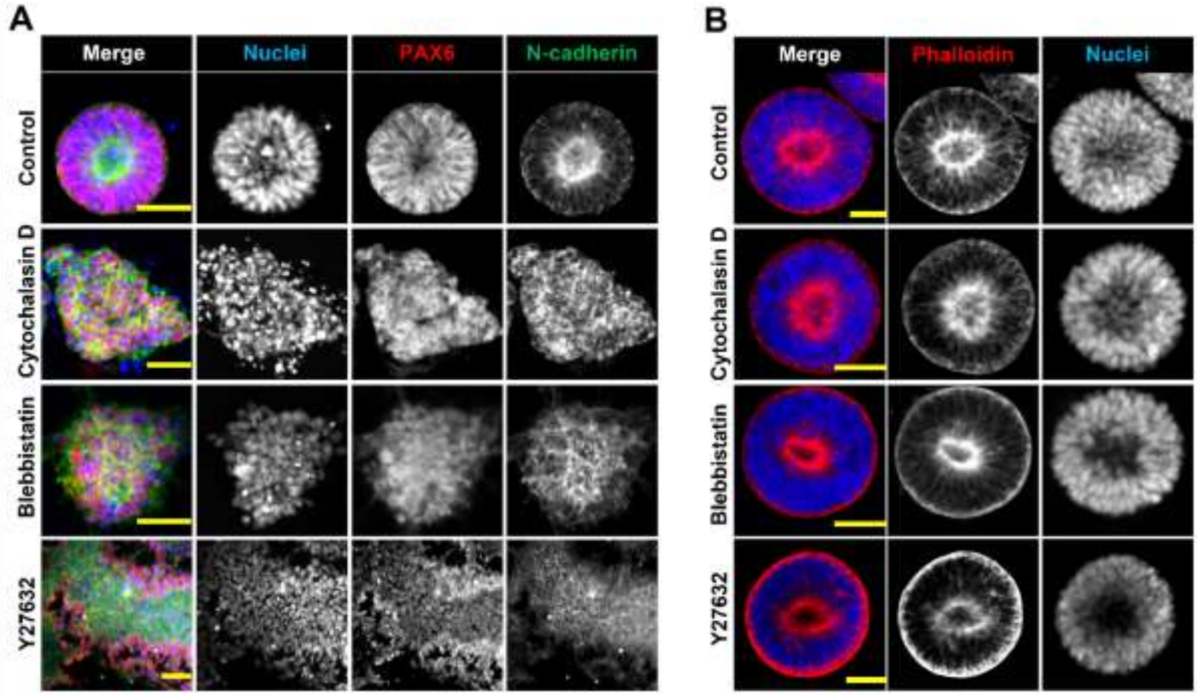

Figure S3. A. Immunofluorescence staining of N-cadherin and PAX6 in NE cysts on day 7. Y276329 (10 µM), (±)-Blebbistatin (10 µM), and Cytochalasin D (0.2 µM) were added during NE cysts culture from day 1 to day 7. B. Immunofluorescence staining of F-actin (phalloidin) in NE cysts on day 7. Y276329 (10 µM), (±)-Blebbistatin (10 µM), and Cytochalasin D (0.2 µM) were added during NE cysts culture from day 1 to day 3. Scale bars: 50 µm

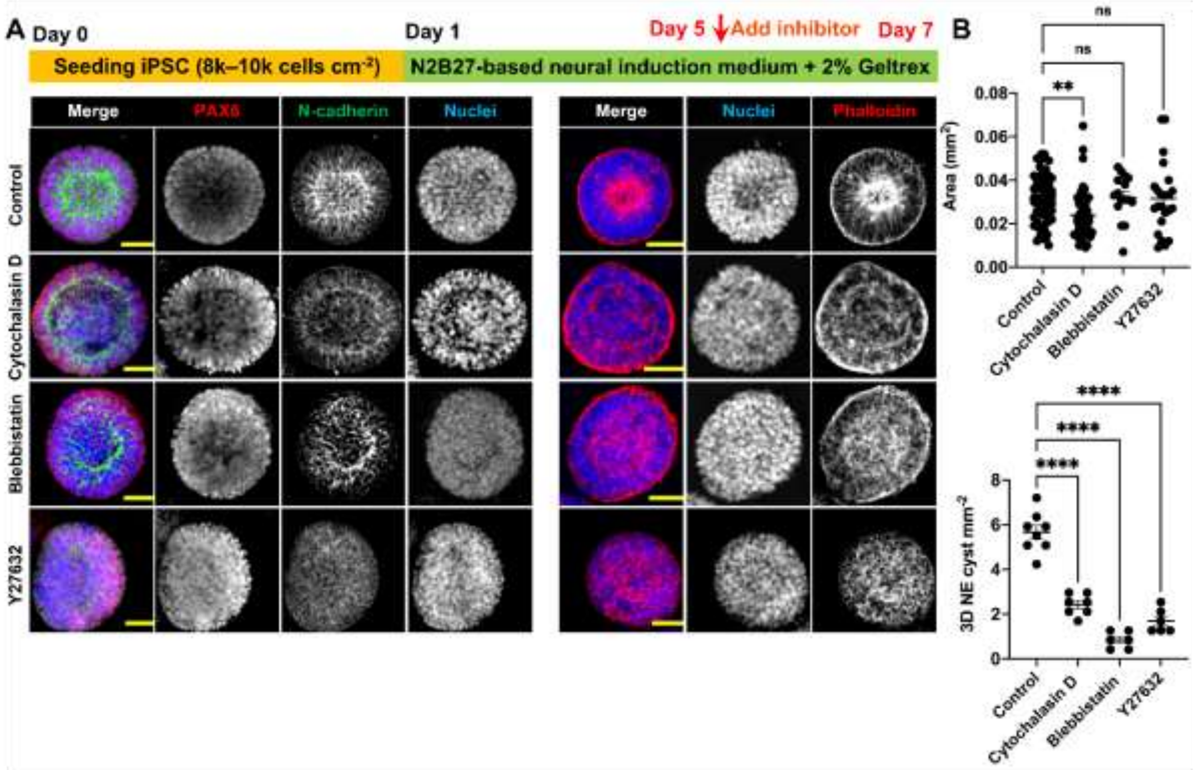

Figure S4. A. Immunofluorescence staining of N-cadherin, PAX6, and F-actin (phalloidin) in NE cysts on day 7. Y276329(10 µM), (±)-Blebbistatin (10 µM), and Cytochalasin D (0.2 µM) were added during NE cysts culture from day 5 to day 7. Scale bars: 50 µm. B. Quantification of number density, area of NE cysts on day 7 under different treatment, n ≥ 6. Error bars represent S.E.M. *P*-values of statistical significance were represented as: * $P < 0.05$, ** $P < 0.01$, *** $P < 0.001$, **** $P < 0.0001$, using One-way analysis of variance (ANOVA) followed by Dunnett multiple comparisons test.

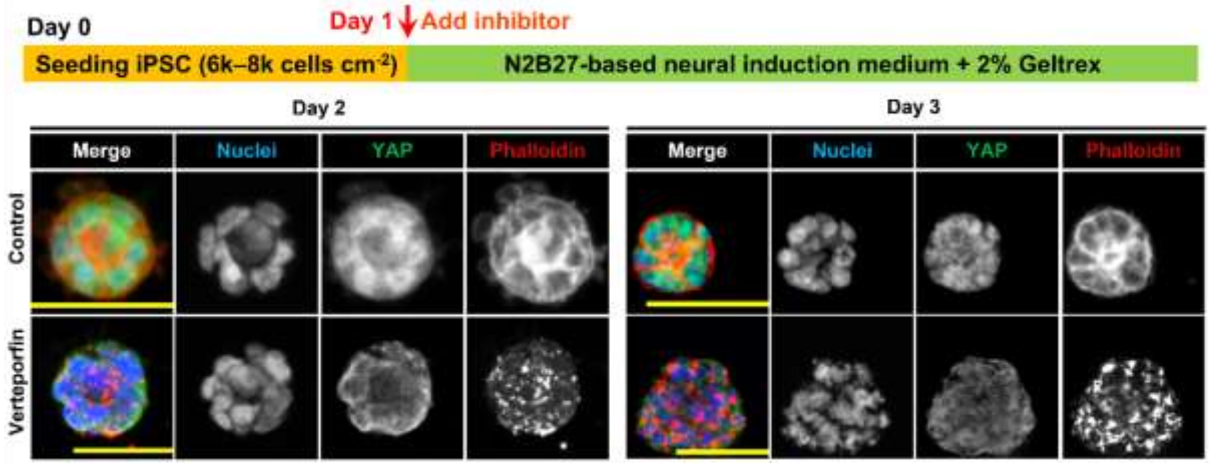

Figure S5. Immunofluorescence staining of YAP and F-actin (Phalloidin) in NE cysts on day 2 and day 3. Verteporfin (1 µM) was added from day 1 to day 3 during NE cyst culture. Scale bars: 50 µm.

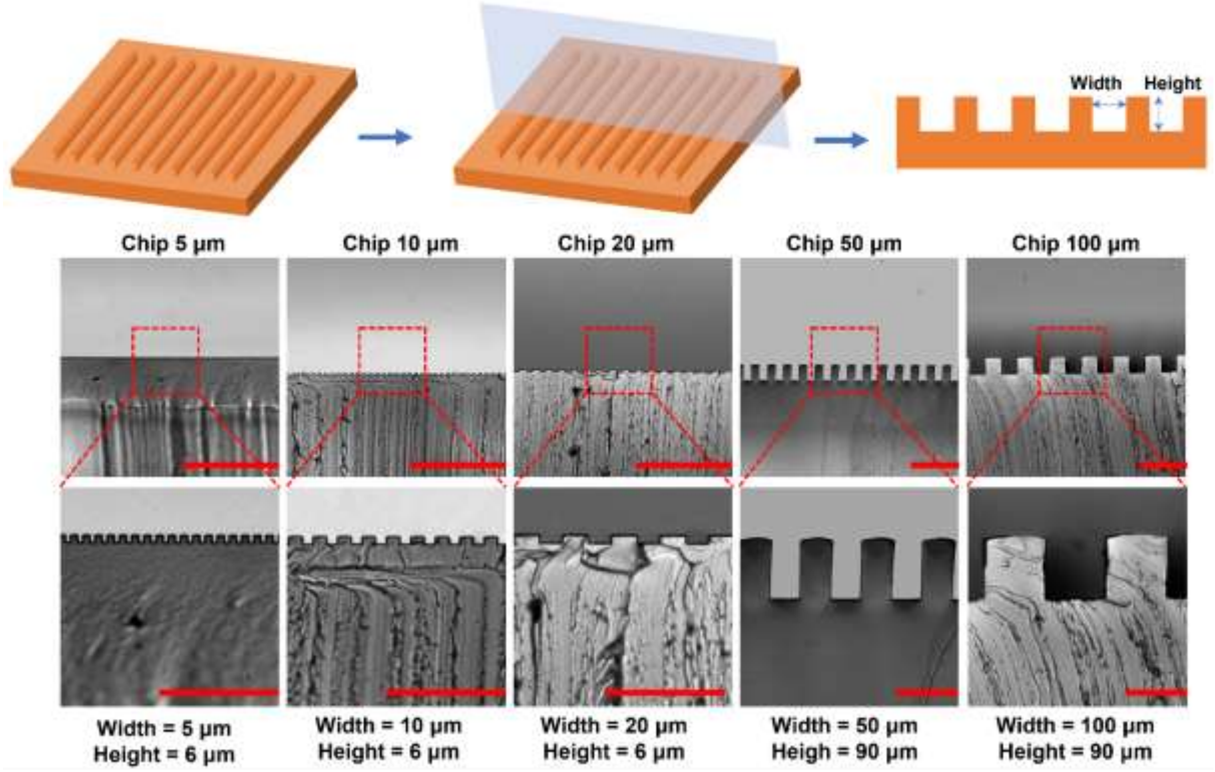

Figure S6. PDMS chips with parallel linear grooves were generated and used for growing NE organoids. Specifically, Chip 5 µm, Chip 10 µm, and Chip 20 µm, containing grooves and ridges with lateral dimensions and spacings of 5 µm, 10 µm, and 20 µm, respectively, and a height of 6 µm, were used for studying the effect of the topographical surface on NE cyst formation. For studying the role of geometrical confinement in NE organoid formation, Chip 50 µm and Chip 100 µm were used, which contain grooves and ridges with lateral dimensions and spacings of 50 µm and 100 µm, respectively, and a height of 90 µm. Flat PDMS substrates were used as a control substrate in all studies. Scale bar: 300 µm (top panels) and 100 µm (bottom panels).

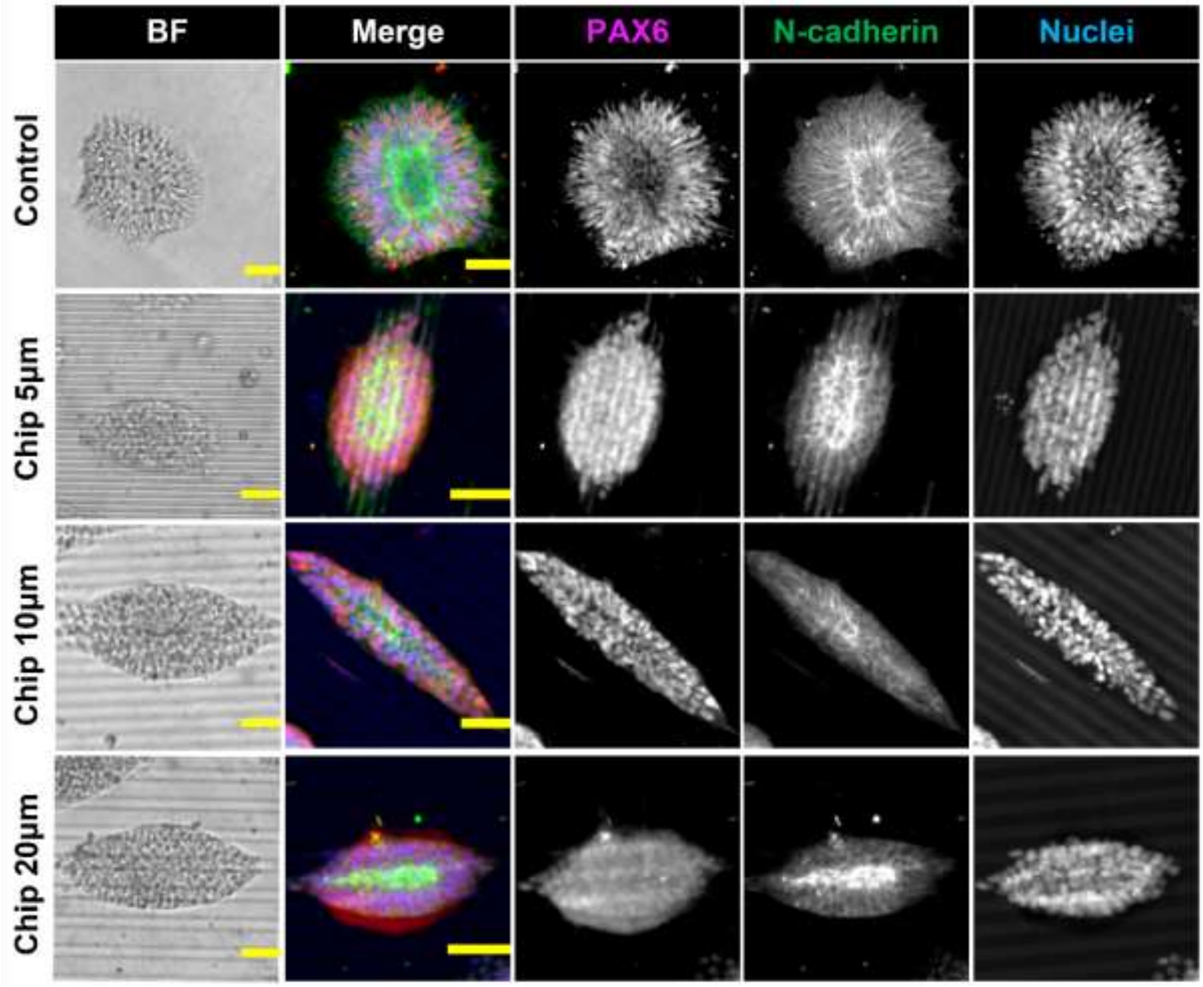

Figure S7. Multicellular structures growing on PDMS chip with linear grooves of different dimensions (Chip 5 μm, Chip 10 μm, and Chip 20 μm) and control PDMS chip with flat surface. Bright-field images and immunofluorescence staining of N-cadherin and PAX6 in multicellular structures on day 7. Scale bars: 50 μm.

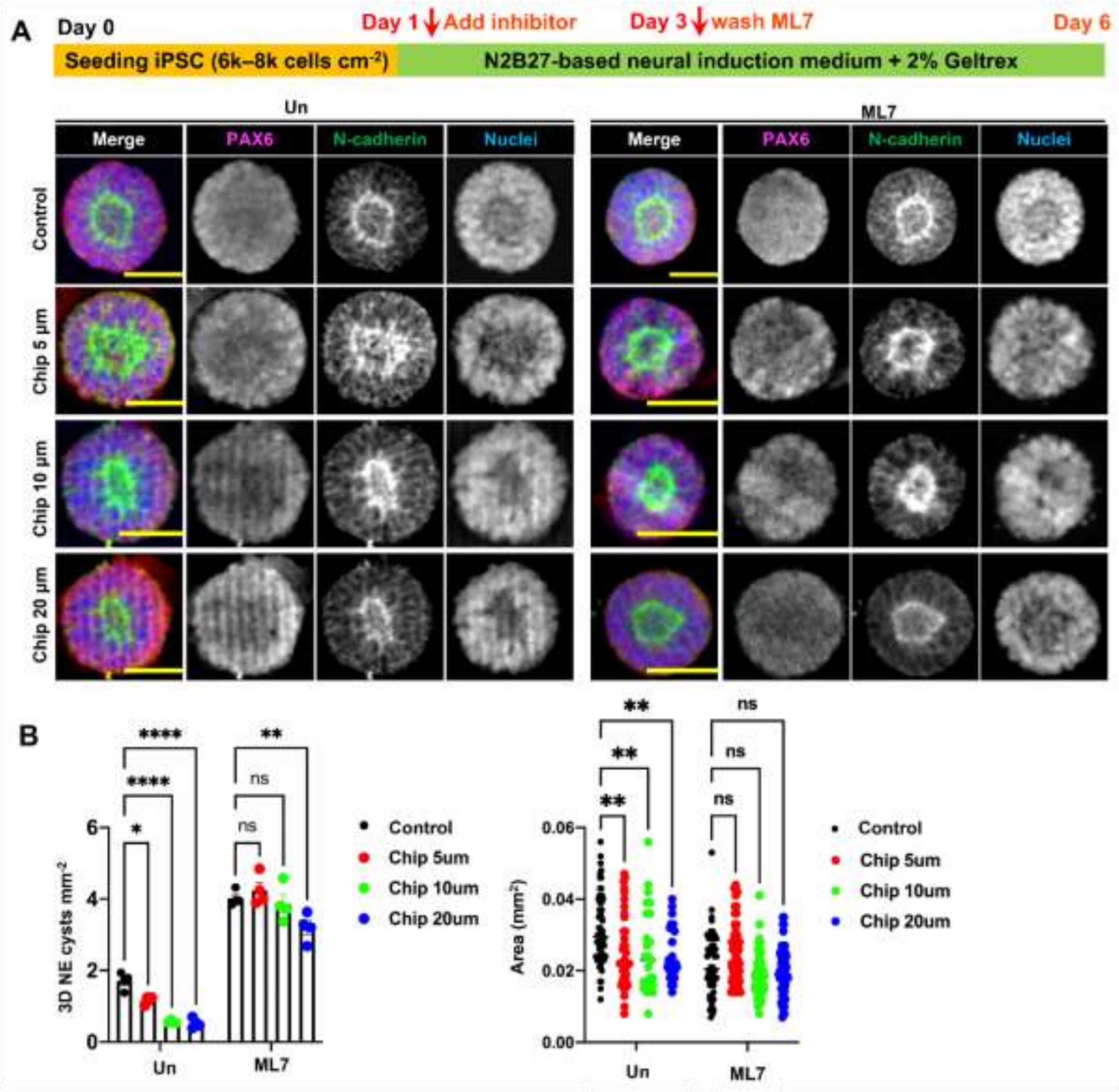

Figure S8. A. Immunofluorescent staining of N-cadherin and PAX6 in NE cysts growing on PDMS chip with linear grooves of different dimensions (Chip 5 µm, Chip 10 µm, and Chip 20 µm) and control PDMS chip with flat surface on day 7. ML7 (10 µM) were added from day 1 to day 3 during NE cyst culture. Scale bars: 50 µm. B. Quantification of number density and area of 3D NE cysts growing on PDMS chip with linear grooves of different dimensions (Chip 5 µm, Chip 10 µm, and Chip 20 µm) and control PDMS chip with flat surface on day 7, $n \geq 3$. Error bars represent S.E.M. *P*-values of statistical significance were represented as: * $P < 0.05$, ** $P < 0.01$, *** $P < 0.001$, **** $P < 0.0001$, using One-way analysis of variance (ANOVA) followed by Dunnett multiple comparisons test.

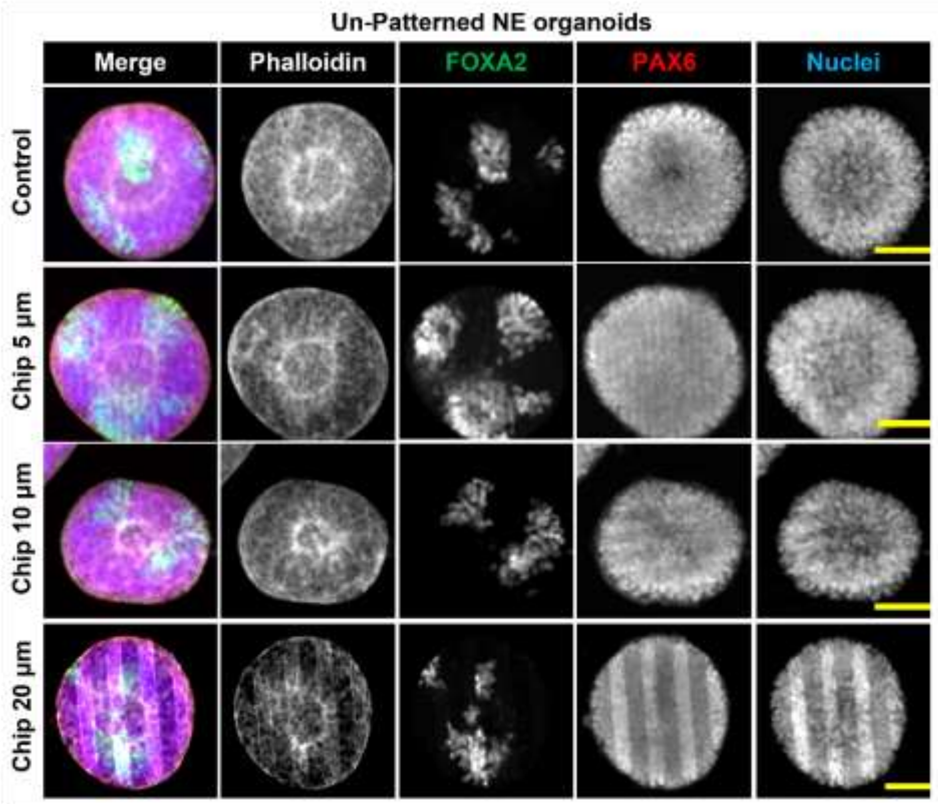

Figure S9. Example NE organoids growing on PDMS chips with linear grooves of different dimensions (Chip 5 µm, Chip 10 µm, Chip 20 µm) and control PDMS chip with flat surface. Immunofluorescence staining of F-actin (phalloidin), FOXA2, and PAX6 in NE organoids on day 18, showing representative examples of unsuccessful floor-plate patterning in NE organoids. Scale bars: 50 µm.

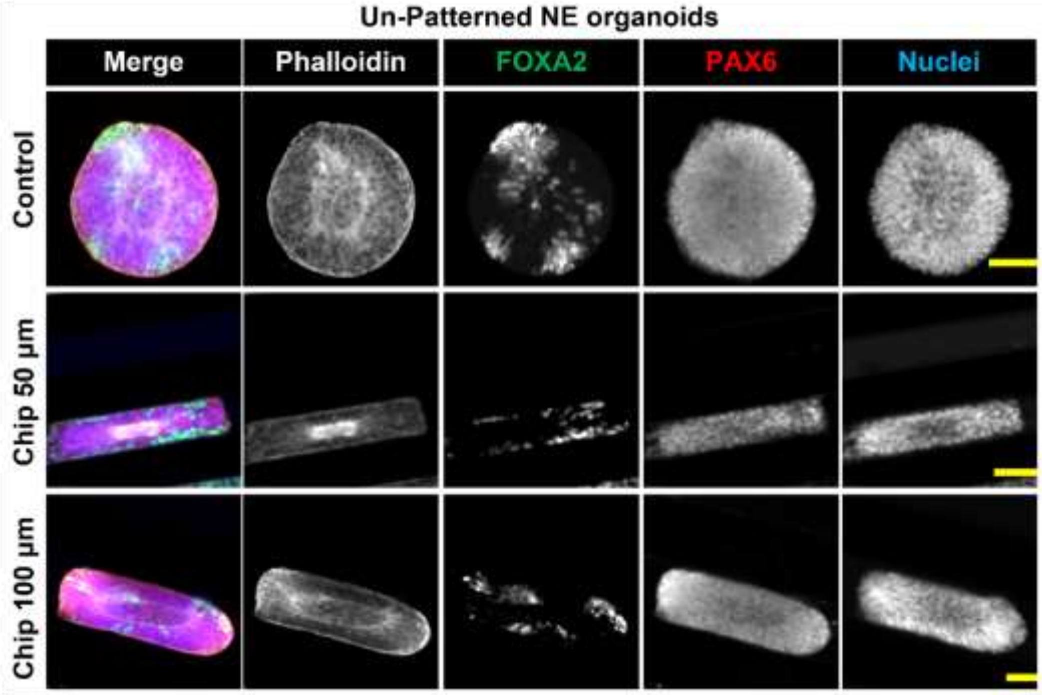

Figure S10. NE organoids growing on PDMS chip with linear grooves micro-niches of different dimensions (Chip 50 µm, Chip 100 µm) and control PDMS chip with flat surface. Immunofluorescence staining of F-actin (phalloidin), FOXA2, and PAX6 in NE organoids on day 18, showing representative examples of unsuccessful floor-plate patterning in NE organoids. Scale bars: 50 µm.
